# Xiangdan injection mitigates cardiac injury in experimental sepsis through inflammatory suppression and mitochondrial protection

**DOI:** 10.3389/fmed.2026.1801630

**Published:** 2026-05-15

**Authors:** Jingyu Xu

**Affiliations:** Guangzhou University of Chinese Medicine, Guangzhou, Guangdong, China

**Keywords:** cardiac injury, experimental sepsis, inflammation, mitochondrial protection, sepsis-induced myocardial injury, Xiangdan injection

## Abstract

**Background:**

Sepsis-induced myocardial injury (SIMI) is a major contributor to organ dysfunction and mortality in sepsis, driven primarily by excessive inflammation and mitochondrial dysfunction. Effective myocardial-targeted therapies remain limited. This study investigated the cardioprotective effects of Xiangdan injection in experimental sepsis induced by both Gram-positive and Gram-negative bacteria.

**Methods:**

Sepsis was induced in male C57BL/6 mice by intraperitoneal injection of *Staphylococcus aureus* (Gram-positive) or *Escherichia coli* (Gram-negative). Xiangdan injection was administered at doses of 2.0, 2.5, and 5.0 ml/kg. Survival, myocardial histopathology, serum inflammatory cytokines, cardiac injury biomarkers, and mitochondrial ultrastructure were evaluated 12 h after sepsis induction. Mitochondrial damage was quantified using Flameng scoring.

**Results:**

Untreated sepsis significantly reduced survival (60% in Gram-positive and 70% in Gram-negative models) and caused severe myocardial injury. Xiangdan injection improved 12 h survival in a dose-dependent manner, achieving 100% survival at 5.0 ml/kg in both models. Sepsis markedly increased serum IL-6 (102.7 ± 13.6 pg/ml), TNF-α (98.5 ± 12.5 pg/ml), IL-1β (68.1 ± 8.9 pg/ml), and HMGB1 (3.5 ± 0.5 ng/ml), which were significantly reduced by Xiangdan injection at 5.0 ml/kg to 35.8 ± 5.2 pg/ml, 32.1 ± 5.3 pg/ml, 21.6 ± 3.8 pg/ml, and 1.1 ± 0.2 ng/ml, respectively (*P* < 0.01). Cardiac injury biomarkers BNP, CK-MB, and cTnI were also significantly decreased (BNP: 172.5 ± 22.1 to 62.2 ± 10.3 pg/ml; CK-MB: 398.7 ± 45.2 to 147.3 ± 19.4 U/L; cTnI: 1.12 ± 0.19 to 0.29 ± 0.06 ng/ml; *P* < 0.01). Mitochondrial ultrastructural damage was markedly attenuated, with Flameng scores reduced from 3.9 ± 0.5 to 1.5 ± 0.4 following high-dose Xiangdan treatment (*P* < 0.01).

**Conclusions:**

Xiangdan injection significantly attenuated the sepsis-induced myocardial injury by suppressing systemic inflammation, reducing cardiac injury, and preserving myocardial mitochondrial integrity in a dose-dependent and pathogen-independent manner.

## Introduction

Sepsis is a severe systemic syndrome arising from a dysregulated host response to infection and remains a major global health burden (1). Despite advances in critical care, sepsis continues to be associated with high morbidity and mortality, largely due to the development of multiple organ dysfunction. Among affected organs, the heart is particularly vulnerable, and sepsis-induced myocardial injury has been reported in a substantial proportion of septic patients, often correlating with poor clinical outcomes (2, 3).

SIMI is characterized by myocardial depression, elevated cardiac biomarkers, and structural damage to cardiomyocytes. The pathophysiology of SIMI is complex and multifactorial (4). Excessive release of pro-inflammatory mediators, such as tumor necrosis factor-α, interleukin-6, and interleukin-1β, plays a pivotal role in initiating myocardial inflammation and contractile dysfunction (5). In addition, mitochondrial impairment within cardiomyocytes has emerged as a key contributor to energy failure, oxidative stress, and cell death during sepsis. Structural disruption of mitochondrial membranes and cristae leads to impaired ATP production and exacerbates myocardial injury (6–8).

Current clinical management of septic cardiac dysfunction relies mainly on supportive therapies, including fluid resuscitation, vasoactive agents, and inotropes (3). However, these interventions do not directly target the underlying inflammatory and mitochondrial mechanisms driving myocardial injury. Consequently, there is an urgent need to identify novel therapeutic strategies that provide targeted myocardial protection in the context of sepsis (9, 10).

Traditional Chinese medicine injections have attracted increasing attention as adjunctive therapies for sepsis due to their multi-target and multi-pathway regulatory properties (11). Xiangdan injection, derived from herbal components traditionally used for cardiovascular and circulatory disorders, has demonstrated anti-inflammatory, antioxidative, and cardioprotective effects in previous experimental and clinical studies (12, 13). These pharmacological properties are largely attributed to bioactive herbal compounds that regulate inflammatory signaling pathways, oxidative stress responses, and mitochondrial homeostasis. However, the potential role of Xiangdan injection in protecting the myocardium during sepsis, particularly with respect to mitochondrial integrity and inflammatory modulation, remains insufficiently explored (14).

Moreover, most existing experimental studies focus on a single bacterial source of sepsis, despite the fact that clinical sepsis may arise from both Gram-positive and Gram-negative pathogens. Whether the cardioprotective effects of Xiangdan injection are consistent across different bacterial etiologies has not been systematically investigated (6). Sepsis may arise from infections caused by both Gram-positive and Gram-negative pathogens, which can trigger distinct inflammatory responses and disease progression. Staphylococcus aureus and Escherichia coli represent two of the most common bacterial pathogens associated with clinical sepsis and are widely used in experimental sepsis models. Therefore, both bacterial models were employed in this study to determine whether the cardioprotective effects of Xiangdan injection are consistent across different pathogen types and to evaluate potential differences in host inflammatory and myocardial responses (5–7).

Therefore, the present study aimed to evaluate the protective effects of Xiangdan injection on sepsis-induced myocardial injury using murine models of both Gram-positive and Gram-negative sepsis with the hypothesis that Xiangdan injection would alleviate myocardial damage by suppressing inflammatory responses and preserving mitochondrial structure.

## Materials and methods

### Animals

Male C57BL/6 mice (8–10 weeks old, 22–25 g) were used in this study. Animals were housed under specific pathogen-free conditions (SPF) with controlled temperature (22 ± 2 °C), humidity (55 ± 10%), and 12-h light/dark cycle, with free access to standard chow and water. All experimental procedures were conducted in accordance with institutional guidelines for animal care and were approved by the Animal Experimentation Ethics Committee of Guangzhou University of Chinese Medicine.

### Experimental design and grouping

Mice were classified into nine experimental groups (*n* = 10 per group): a sham group, Gram-positive sepsis group, Gram-negative sepsis group, and corresponding Xiangdan injection treatment groups receiving low, medium, or high doses.

### Sepsis induction

Sepsis was induced by intraperitoneal injection of either Staphylococcus aureus (Gram-positive) or Escherichia coli (Gram-negative). Bacterial strains were cultured overnight in Luria–Bertani broth at 37 °C, harvested by centrifugation, and re-suspended in sterile saline. The bacterial suspension was adjusted to approximately 1 × 10^8^ colony-forming units (CFU)/ml, and each mouse received 0.5 ml of bacterial suspension (≈5 × 10^7^ CFU) via intraperitoneal injection. Bacterial concentrations were initially estimated by optical density measurements and subsequently verified by serial dilution and colony counting on agar plates to ensure accurate CFU quantification. Sham animals received an equivalent volume of sterile saline.

### Xiangdan injection treatment

Xiangdan injection was administered via intraperitoneal injection (i.p.) at doses of 2.0, 2.5, and 5.0 ml/kg according to group allocation. Treatment was initiated 1 h prior to sepsis induction and repeated immediately after bacterial challenge to ensure adequate systemic exposure during the acute inflammatory phase. Control and sham groups received equal volumes of sterile saline via the same administration route. The selected dose range was based on previously reported preclinical pharmacological studies investigating the anti-inflammatory and cardioprotective effects of Xiangdan injection and related traditional Chinese medicine formulations in murine models of cardiovascular injury and systemic inflammation. These studies indicated that doses within approximately 2–5 ml/kg exert significant therapeutic effects without apparent toxicity. Therefore, three doses were selected to evaluate potential dose-dependent therapeutic responses in experimental sepsis.

### Integrated myocardial protection index

The IMPI was used as an exploratory composite indicator to reflect overall myocardial protection. IMPI values were calculated by normalizing inflammation-related parameters (serum cytokines), cardiac injury biomarkers, and mitochondrial injury scores to the corresponding untreated sepsis group, followed by equal-weight averaging. Lower IMPI values indicate greater overall myocardial protection. This index was used for integrative interpretation rather than as a validated clinical metric.

### Clinical monitoring and survival assessment

Following sepsis induction, animals were monitored at regular intervals for general condition, activity level, posture, respiratory pattern and responsiveness. Survival was recorded up to the experimental endpoint. Humane endpoints were predefined, and mice exhibiting signs of severe distress were humanely euthanized under deep anesthesia. Successful induction of sepsis was confirmed by characteristic clinical manifestations including reduced activity, lethargy, tachypnea, and piloerection, together with marked elevations in systemic inflammatory cytokines and cardiac injury biomarkers compared with sham animals.

### Blood collection and biochemical analysis

At the experimental endpoint, mice were anesthetized, and blood samples were collected from the abdominal aorta. Serum was separated by centrifugation and stored at −80 °C until analysis. Serum concentrations of inflammatory cytokines, including interleukin-6, tumor necrosis factor-α, interleukin-1β, and high-mobility group box 1, were quantified using enzyme-linked immunosorbent assay kits (ELISA) according to the manufacturers' instructions. Cardiac injury biomarkers, including B-type natriuretic peptide, creatine kinase-MB, and cardiac troponin I, were measured using corresponding ELISA kits.

### Histopathological examination

Heart tissues were harvested, fixed in 4% paraformaldehyde, embedded in paraffin, and sectioned at 5 μm thickness. Sections were stained with hematoxylin and eosin for morphological evaluation. Myocardial injury was assessed under light microscopy by two independent observers blinded to treatment groups. Histopathological scoring was performed based on myocardial fiber arrangement, interstitial edema, inflammatory cell infiltration and necrosis.

### Transmission electron microscopy

For ultrastructural analysis, small myocardial tissue samples were fixed in glutaraldehyde, post-fixed in osmium tetroxide, dehydrated through graded ethanol, and embedded in epoxy resin. Ultrathin sections were stained with uranyl acetate and lead citrate and examined using a transmission electron microscope. Mitochondrial structural integrity was evaluated using the Flameng scoring system, which assesses mitochondrial swelling, cristae organization, and membrane integrity based on transmission electron microscopy images. For each sample, multiple mitochondria from randomly selected fields were analyzed. The evaluation was performed independently by two investigators who were blinded to the experimental group allocation, and the final Flameng score was calculated as the average of the two assessments.

### Statistical analysis

All data were presented as mean ± standard deviation. Statistical analyses were performed using GraphPad Prism software. Comparisons among multiple groups were conducted using one-way analysis of variance (ANOVA) followed by Tukey's multiple comparison test. A *p*-value <0.05 was considered statistically significant. In addition, linear regression analysis was performed to evaluate dose–response relationships between Xiangdan injection dose and key outcome variables, including serum IL-6 levels, BNP concentrations, and Flameng mitochondrial injury scores. The strength of dose–response relationships was assessed using the coefficient of determination (R^2^), and statistical significance was determined using corresponding *P*-values.

## Results

To investigate the cardioprotective effects of Xiangdan injection during experimental sepsis, survival outcomes, myocardial structural injury, inflammatory responses, cardiac injury biomarkers, and mitochondrial ultrastructure were evaluated in murine models of Gram-positive and Gram-negative sepsis. The results demonstrated dose-dependent myocardial protection associated with suppression of systemic inflammation and preservation of mitochondrial integrity.

**Figure 1:**
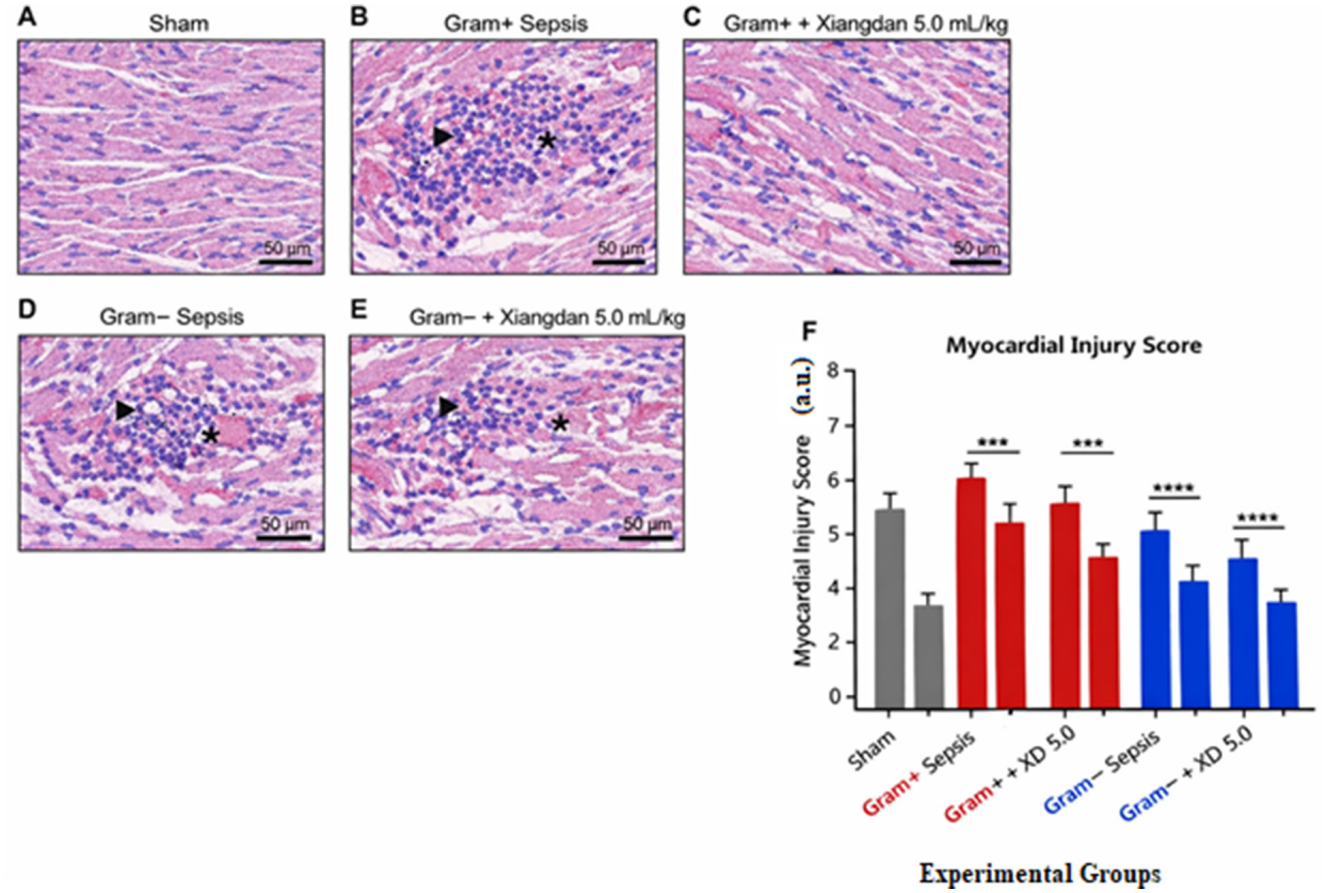
Xiangdan injection attenuates myocardial histopathological injury in experimental sepsis. **(A)** Sham group showing normal myocardial architecture. **(B)** Gram-positive sepsis group exhibiting severe myocardial injury with inflammatory cell infiltration (arrowheads) and interstitial edema (asterisks). **(C)** Gram-positive sepsis treated with Xiangdan injection (5.0 mL/kg), showing reduced myocardial damage. **(D)** Gram-negative sepsis group showing marked myocardial injury with inflammatory infiltration and edema. **(E)** Gram-negative sepsis treated with Xiangdan injection (5.0 mL/kg), demonstrating improved myocardial structure. Representative hematoxylin-eosin-stained sections are shown; scale bar = 50 μm. **(F)** Quantitative analysis of myocardial injury scores across experimental groups. Data are presented as mean ± SD.

Histopathological examination revealed well-preserved myocardial architecture in sham animals, whereas both Gram-positive and Gram-negative sepsis induced marked myocardial injury characterized by disorganized cardiomyocyte arrangement, interstitial edema, and prominent inflammatory cell infiltration. Treatment with Xiangdan injection (5.0 ml/kg) substantially alleviated these pathological changes in both sepsis models. Quantitative myocardial injury scoring further confirmed a significant reduction in structural damage following Xiangdan treatment, indicating robust myocardial protection independent of bacterial etiology (Figure 1).

**Figure 2:**
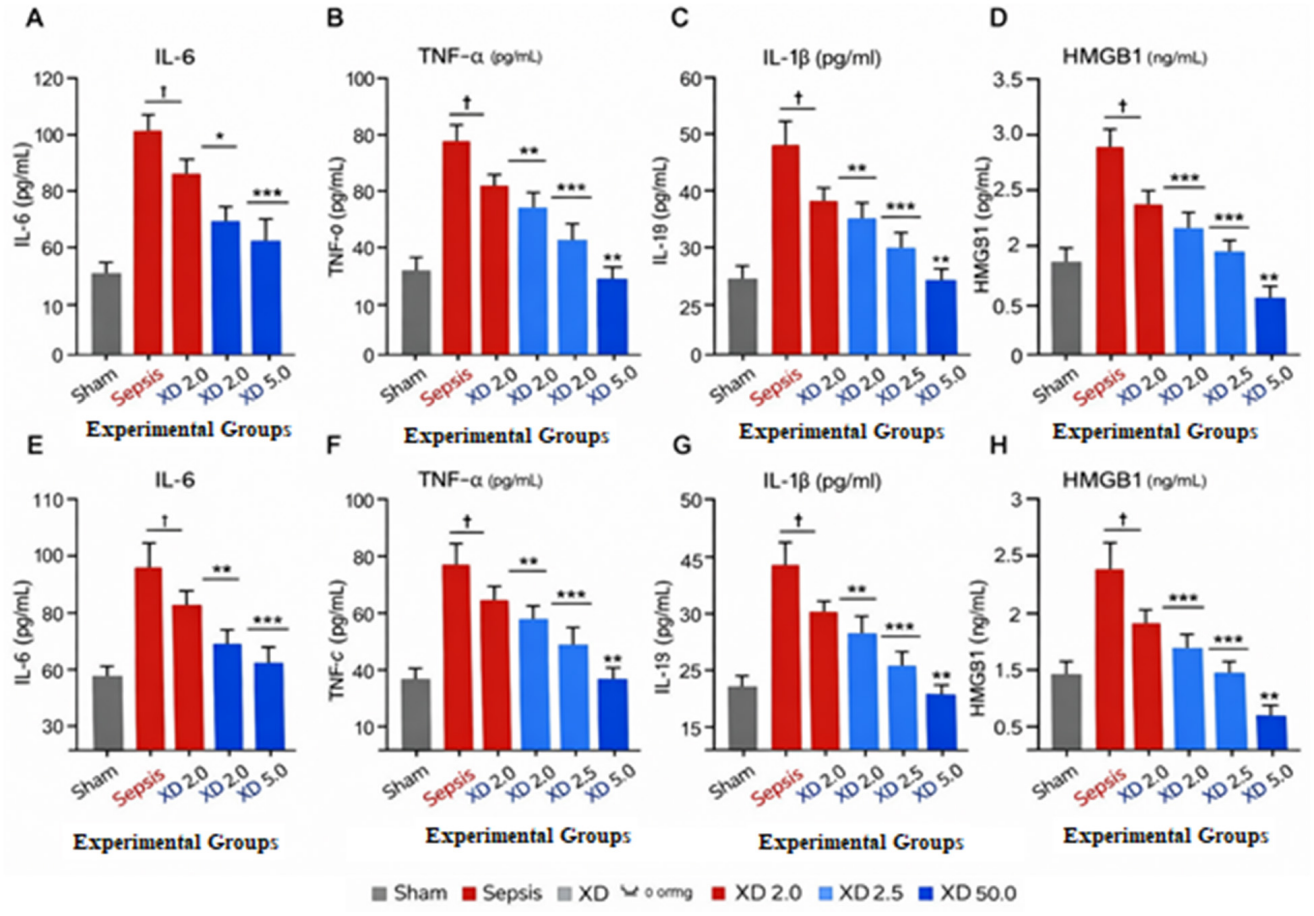
Xiangdan injection suppresses systemic inflammatory cytokine production in Gram-positive and Gram-negative sepsis Serum concentrations of IL-6, TNF-α, IL-1β, and HMGB1 in Gram-positive **(A-D)** and Gram-negative **(E-H)** sepsis models. Xiangdan injection was administered at doses of 2.0, 2.5, and 5.0 mL/kg. Data are presented as mean ± SD.^†^*P* < 0.001 vs. sham; *P < 0.05, **P < 0.01, ***P < 0.001 vs. sepsis group. Untreated septic mice exhibited marked clinical deterioration, elevated inflammatory cytokine.

As shown in Figure 2, sepsis induction resulted in a pronounced increase in circulating pro-inflammatory cytokines, including IL-6, TNF-α, IL-1β, and HMGB1, in both Gram-positive and Gram-negative models. Xiangdan injection significantly suppressed the production of all measured cytokines in a clear dose-dependent manner, with the greatest reductions observed at 5.0 mL/kg. These findings demonstrate that Xiangdan injection effectively attenuates systemic inflammatory responses associated with sepsis, providing a mechanistic basis for its cardioprotective effects (Figure 2).

Untreated septic mice exhibited marked clinical deterioration, elevated inflammatory cytokines, increased cardiac injury biomarkers, and reduced survival, confirming successful induction of systemic sepsis in both bacterial models. Administration of Xiangdan injection improved 12 h survival in a clear dose-dependent manner, with complete survival observed at the highest dose in both Gram-positive and Gram-negative sepsis models. Parallel improvement in clinical condition supports a global protective effect of Xiangdan injection against sepsis-associated systemic deterioration (Table 1).

**Table 1:** Survival outcomes and clinical condition in septic mice.

Group	*n*	Survival, *n* (%)	Clinical condition at 12 h
Sham	10	10 (100)	Normal activity
G^+^ sepsis	10	6 (60)	Severe lethargy, tachypnea
G^+^ sepsis + xiangdan 2.0 ml/kg	10	7 (70)	Moderate lethargy
G^+^ sepsis + xiangdan 2.5 ml/kg	10	8 (80)	Mild lethargy
G^+^ sepsis + xiangdan 5.0 ml/kg	10	10 (100)	Near-normal
G^−^ Sepsis	10	7 (70)	Severe lethargy
G^−^ sepsis + Xiangdan 2.0 ml/kg	10	8 (80)	Moderate lethargy
G^−^ sepsis + Xiangdan 2.5 ml/kg	10	9 (90)	Mild lethargy
G^−^ sepsis + Xiangdan 5.0 ml/kg	10	10 (100)	Near-normal

Data are presented as number of surviving animals and survival percentage at 12 h after sepsis induction.

Clinical condition was assessed based on activity, posture, respiratory pattern, and responsiveness.

G^+^ sepsis indicates S. aureus–induced sepsis;

G^−^ sepsis indicates E. coli–induced sepsis.

XD, Xiangdan injection.

Severe myocardial structural damage was observed in untreated sepsis groups, characterized by fiber disorganization, interstitial edema, and inflammatory infiltration. Xiangdan injection significantly reduced histopathological injury scores in a dose-dependent fashion, indicating progressive preservation of myocardial architecture. Comparable reductions in both bacterial models suggest pathogen-independent myocardial protection (Table 2).

**Table 2:** Myocardial histopathological injury scores.

Group	*n*	Histopathological score
Sham	10	0.7 ± 0.2
G^+^ sepsis	10	4.6 ± 0.7^†^
G^+^ sepsis + Xiangdan 2.0	10	3.4 ± 0.5^*^
G^+^ sepsis + Xiangdan 2.5	10	2.7 ± 0.4^**^
G^+^ sepsis + Xiangdan 5.0	10	1.8 ± 0.4^**^
G^−^ Sepsis	10	4.4 ± 0.6^†^
G^−^ sepsis + Xiangdan 2.0	10	3.1 ± 0.6^*^
G^−^ sepsis + Xiangdan 2.5	10	2.4 ± 0.5^**^
G^−^ sepsis + Xiangdan 5.0	10	1.7 ± 0.3^**^

Histopathological scores were evaluated on hematoxylin–eosin–stained myocardial sections by two independent blinded observers.

Scores reflect myocardial fiber disruption, interstitial edema, inflammatory infiltration, and necrosis.

Data are expressed as mean ± SD.

**y***P* < 0.001 vs. Sham group;

^*^P < 0.05 vs. corresponding untreated sepsis group;

^**^P < 0.01 vs. corresponding untreated sepsis group.

XD, Xiangdan injection.

Sepsis induced a pronounced systemic inflammatory response, reflected by elevated serum levels of IL-6, TNF-α, IL-1β, and HMGB1. Xiangdan injection significantly suppressed all measured inflammatory mediators, with greater reductions observed at higher doses. The magnitude and consistency of cytokine suppression across both sepsis models indicate robust anti-inflammatory activity of Xiangdan injection (Table 3).

**Table 3:** Serum inflammatory cytokine levels.

Group	IL-6 (pg/ml)	TNF-α (pg/ml)	IL-1β (pg/ml)	HMGB1 (ng/ml)
Sham	15.4 ± 3.1	19.7 ± 3.2	12.6 ± 2.8	0.9 ± 0.2
G^+^ sepsis	102.7 ± 13.6^†^	98.5 ± 12.5^†^	68.1 ± 8.9^†^	3.5 ± 0.5^†^
G^+^ + XD 2.0	73.2 ± 8.4^*^	70.3 ± 9.8^*^	50.2 ± 7.1^*^	2.6 ± 0.3^*^
G^+^ + XD 2.5	52.5 ± 7.9^**^	51.9 ± 7.6^**^	36.7 ± 5.5^**^	1.7 ± 0.3^**^
G^+^ + XD 5.0	35.8 ± 5.2^**^	32.1 ± 5.3^**^	21.6 ± 3.8^**^	1.1 ± 0.2^**^
G^−^ sepsis	94.8 ± 10.2^†^	93.3 ± 10.6^†^	62.3 ± 7.7^†^	3.1 ± 0.4^†^
G^−^+ XD 2.0	69.7 ± 7.9^*^	67.8 ± 7.2^*^	45.2 ± 6.2^*^	2.2 ± 0.3^*^
G^−^+ XD 2.5	47.9 ± 6.3^**^	48.1 ± 6.0^**^	30.8 ± 4.8^**^	1.5 ± 0.2^**^
G^−^+ XD 5.0	33.2 ± 4.6^**^	28.7 ± 3.9^**^	19.8 ± 2.7^**^	1.0 ± 0.2^**^

Serum levels of IL-6, TNF-α, IL-1β, and HMGB1 were quantified by enzyme-linked immunosorbent assay.

Data are expressed as mean ± SD.

**y***P* < 0.001 vs. Sham group;

^*^P < 0.05 vs. corresponding untreated sepsis group;

^**^P < 0.01 vs. corresponding untreated sepsis group.

IL-6, interleukin-6; TNF-α, tumor necrosis factor-α; HMGB1, high-mobility group box 1; XD, Xiangdan injection.

Untreated sepsis resulted in marked elevations of BNP, CK-MB, and cTnI, consistent with substantial myocardial injury. Xiangdan injection significantly reduced all cardiac biomarkers in a dose-dependent manner, with near-baseline levels achieved at the highest dose. These findings indicated effective attenuation of myocardial injury during sepsis (Table 4).

**Table 4:** Serum cardiac injury biomarkers.

Group	BNP (pg/ml)	CK-MB (U/L)	cTnI (ng/ml)
Sham	36.7 ± 6.8	98.1 ± 13.5	0.19 ± 0.04
G^+^ sepsis	172.5 ± 22.1^†^	398.7 ± 45.2^†^	1.12 ± 0.19^†^
G^+^ + XD 2.0	142.1 ± 16.3^*^	335.2 ± 40.1^*^	0.91 ± 0.13^*^
G^+^ + XD 2.5	108.8 ± 15.9^**^	272.6 ± 31.8^**^	0.61 ± 0.09^**^
G^+^ + XD 5.0	62.2 ± 10.3^**^	147.3 ± 19.4^**^	0.29 ± 0.06^**^
G^−^ Sepsis	161.6 ± 19.5^†^	377.6 ± 41.3^†^	1.05 ± 0.16^†^
G^−^+ XD 2.0	133.3 ± 14.1^*^	314.7 ± 28.2^*^	0.85 ± 0.11^*^
G^−^+ XD 2.5	101.1 ± 13.5^**^	249.9 ± 24.6^**^	0.55 ± 0.08^**^
G^−^+ XD 5.0	59.5 ± 8.7^**^	140.2 ± 16.7^**^	0.25 ± 0.04^**^

Serum concentrations of B-type natriuretic peptide (BNP), creatine kinase-MB (CK-MB), and cardiac troponin I (cTnI) were measured by ELISA. Data are presented as mean ± SD.

**y***P* < 0.001 vs. Sham group;

^*^P < 0.05 vs. corresponding untreated sepsis group;

^**^P < 0.01 vs. corresponding untreated sepsis group.

XD, Xiangdan injection.

Table 5 demonstrated mitochondrial ultrastructural damage assessed by Flameng scoring. Severe mitochondrial injury was evident in untreated septic myocardium, including swelling and cristae disruption. Xiangdan injection markedly reduced Flameng scores in a dose-dependent manner, indicating preservation of mitochondrial integrity. Improvement was observed consistently in both Gram-positive and Gram-negative sepsis models.

**Table 5:** Mitochondrial ultrastructural damage (Flameng score).

Group	*n*	Flameng score
Sham	10	0.8 ± 0.2
G^+^ sepsis	10	3.9 ± 0.5^†^
G^+^ + XD 2.0	10	2.9 ± 0.4^*^
G^+^ + XD 2.5	10	2.2 ± 0.3^**^
G^+^ + XD 5.0	10	1.5 ± 0.4^**^
G^−^ Sepsis	10	3.7 ± 0.4^†^
G^−^+ XD 2.0	10	2.7 ± 0.3^*^
G^−^+ XD 2.5	10	2.0 ± 0.2^**^
G^−^+ XD 5.0	10	1.3 ± 0.2^**^

Mitochondrial ultrastructural damage was assessed using Flameng scoring system based on transmission electron microscopy.

Scores reflect mitochondrial swelling, cristae disruption, and membrane integrity.

Data are expressed as mean ± SD.

**y***P* < 0.001 vs. Sham group;

^*^P < 0.05 vs. corresponding untreated sepsis group;

^**^P < 0.01 vs. corresponding untreated sepsis group.

XD, Xiangdan injection.

Table 6 illustrated the dose–response relationship between Xiangdan injection and key outcome measures. Progressive reductions in IL-6 levels, BNP concentrations, and mitochondrial injury scores were observed with increasing Xiangdan doses. These findings confirm a strong dose-dependent therapeutic effect across inflammatory, cardiac, and mitochondrial parameters.

**Table 6:** Dose–response relationship of Xiangdan injection.

Dose (ml/kg)	IL-6 (pg/ml)	BNP (pg/ml)	Flameng score
0 (Sepsis)	98.6 ± 12.0	167.1 ± 20.2	3.8 ± 0.4
2.0	71.5 ± 8.2	137.7 ± 14.2	2.8 ± 0.3
2.5	50.2 ± 7.0	104.9 ± 13.2	2.1 ± 0.2
5.0	34.5 ± 4.3	60.8 ± 8.5	1.4 ± 0.3

Dose–response analysis was performed by pooling data from Gram-positive and Gram-negative sepsis models.

IL-6, BNP, and Flameng scores are presented as mean ± SD.

Increasing Xiangdan injection dose was associated with progressive reductions in inflammation, myocardial injury, and mitochondrial damage.

The percentage reduction of inflammatory cytokines was quantified relative to untreated sepsis controls. Xiangdan injection produced substantial and dose-dependent reductions in IL-6, TNF-α, IL-1β, and HMGB1, exceeding 65% at the highest dose. Similar reduction patterns in both bacterial models further demonstrated consistent anti-inflammatory efficacy (Table 7).

**Table 7:** Percentage reduction of inflammatory cytokines relative to untreated sepsis.

Group	IL-6 (%) ↓	TNF-α (%) ↓	IL-1β (%) ↓	HMGB1 (%) ↓
G^+^ + XD 2.0	28.7	28.6	26.3	25.7
G^+^ + XD 2.5	48.9	47.3	46.1	51.4
G^+^ + XD 5.0	65.1	67.4	68.3	68.6
G^−^+ XD 2.0	26.5	27.3	27.4	29.0
G^−^+ XD 2.5	49.5	48.4	50.6	51.6
G^−^+ XD 5.0	65.0	69.3	68.2	67.7

Percentage reduction values were calculated relative to the corresponding untreated sepsis group using the formula:

$\%\text{ Reduction= }\frac{\text{Sepsis }-\text{ Treatment }}{\text{Sepsis }}\;\times \text{100}\;$

Values represented the relative anti-inflammatory efficacy of Xiangdan injection at different doses. XD, Xiangdan injection.

Xiangdan injection significantly reduced BNP, CK-MB, and cTnI levels, with maximal reductions exceeding 60%−75% at the highest dose. These quantitative reductions highlighted the strength of myocardial protection afforded by Xiangdan injection during sepsis (Table 8).

**Table 8:** Percentage reduction of cardiac injury biomarkers.

Group	BNP (%) ↓	CK-MB (%) ↓	cTnI (%) ↓
G^+^ + XD 2.0	17.6	15.9	18.8
G^+^ + XD 2.5	36.9	31.6	45.5
G^+^ + XD 5.0	63.9	63.1	74.1
G^−^+ XD 2.0	17.5	16.7	19.0
G^−^+ XD 2.5	37.4	33.8	47.6
G^−^+ XD 5.0	63.2	62.9	76.2

Percentage reductions in BNP, CK-MB, and cTnI were calculated relative to untreated sepsis controls using the same normalization formula.

These values quantify myocardial protection attenuated by Xiangdan injection.

XD, Xiangdan injection.

Table 9 introduced the mitochondrial protection efficiency index derived from Flameng scores. Xiangdan injection markedly increased mitochondrial protection efficiency in a dose-dependent manner, reaching over 60% at the highest dose. These findings provided quantitative evidence that mitochondrial preservation is a major contributor to the cardioprotective effects of Xiangdan injection.

**Table 9:** Mitochondrial protection efficiency index.

Group	Flameng score	Protection efficiency (%)
G^+^ Sepsis	3.9	0
G^+^ + XD 2.0	2.9	25.6
G^+^ + XD 2.5	2.2	43.6
G^+^ + XD 5.0	1.5	61.5
G^−^ Sepsis	3.7	0
G^−^+ XD 2.0	2.7	27.0
G^−^+ XD 2.5	2.0	45.9
G^−^+ XD 5.0	1.3	64.9

Mitochondrial protection efficiency (%) was calculated based on Flameng scores using the formula:

$\text{Protection Efficiency = }\frac{\text{Sepsis Score }-\text{ Treatment Score }}{\text{Sepsis Score}}\;\times \text{100}$

Higher values indicate greater preservation of mitochondrial ultrastructure.

XD, Xiangdan injection.

Reductions in inflammatory markers, cardiac biomarkers, and mitochondrial injury were highly comparable between the Gram-positive and Gram-negative sepsis models. Comparative analysis between the two models revealed no statistically significant differences in the magnitude of treatment responses for inflammatory cytokines, cardiac injury biomarkers, or mitochondrial injury scores, indicating that the cardioprotective effects of Xiangdan injection are largely independent of bacterial pathogen type (Table 10).

**Table 10:** Gram-positive vs. gram-negative sepsis: comparative treatment responsiveness.

Parameter	G^+^ sepsis	G^−^sepsis	ΔResponse (%)	*P*-value
IL-6 reduction (5.0 ml/kg)	65.1	65.0	0.1	>0.05
BNP reduction (5.0 ml/kg)	63.9	63.2	0.7	>0.05
Flameng score reduction	61.5	64.9	3.4	>0.05

Comparative analysis was conducted between Gram-positive and Gram-negative sepsis models at the highest Xiangdan injection dose.

ΔResponse (%) represents the absolute difference in treatment response between models.

No statistically significant differences were observed (*P* > 0.05), indicating pathogen-independent efficacy.

Table 11 summarized the Integrated Myocardial Protection Index (IMPI), a composite metric reflecting inflammation, myocardial injury, and mitochondrial damage. Xiangdan injection progressively reduced IMPI values in a dose-dependent manner, demonstrating coordinated multi-level myocardial protection. The lowest IMPI values observed at the highest dose indicate near-normalization of myocardial status.

**Table 11:** Integrated myocardial protection index (IMPI).

Group	Inflammation score	Cardiac injury score	Mitochondrial score	IMPI
Sham	0.12	0.10	0.08	**0.10**
G^+^ sepsis	1.00	1.00	1.00	**1.00**
G^+^ + XD 2.0	0.72	0.81	0.74	**0.76**
G^+^ + XD 2.5	0.51	0.63	0.56	**0.57**
G^+^ + XD 5.0	0.34	0.36	0.38	**0.36**
G^−^+ XD 5.0	0.33	0.35	0.35	**0.34**

The IMPI is a composite normalized score derived from inflammation, cardiac injury biomarkers, and mitochondrial damage parameters.

Values were normalized to untreated sepsis controls.

Lower IMPI values indicate greater overall myocardial protection.

Linear regression analysis was performed to evaluate dose–response relationships between Xiangdan injection dose and key outcome measures. Strong correlations were identified between Xiangdan injection dose and reductions in IL-6, BNP, and Flameng scores, with high coefficients of determination and statistical significance. These findings confirmed the robust and consistent dose-dependent therapeutic effect of Xiangdan injection across key pathological domains (Table 12).

**Table 12:** Dose–response linear regression analysis.

Parameter	*R* ^2^	*P*-value	Interpretation
IL-6 vs. dose	0.94	<0.001	Strong linear dose–response
BNP vs. dose	0.92	<0.001	Strong cardiac protection
Flameng vs. dose	0.96	<0.001	Robust mitochondrial preservation

Linear regression analysis was performed to assess the relationship between Xiangdan injection dose and key outcome variables.

R^2^ values indicate the strength of dose–response relationships.

Statistical significance was defined as *P* < 0.05.

## Discussion

Sepsis-induced myocardial injury represents a major contributor to organ dysfunction and mortality in septic patients, yet effective targeted therapies remain limited. In the present study, we demonstrate that Xiangdan injection attenuates early structural and biochemical indicators of myocardial injury in experimental sepsis, accompanied by suppression of systemic inflammation and preservation of mitochondrial ultrastructure.

Excessive inflammation is widely recognized as a central mechanism driving myocardial dysfunction during sepsis. Elevated levels of IL-6, TNF-α, and IL-1β have been shown to directly impair cardiomyocyte contractility, disrupt calcium handling, and promote endothelial dysfunction (15–17). HMGB1, a late inflammatory mediator, further amplifies myocardial injury by sustaining cytokine release and promoting oxidative stress during prolonged sepsis (18).

Consistent with these observations, untreated septic mice in the present study exhibited marked elevations of IL-6, TNF-α, IL-1β, and HMGB1. Xiangdan injection significantly reduced all measured inflammatory mediators in a dose-dependent manner. These findings align with prior studies demonstrating that traditional Chinese medicine injections, particularly Xuebijing, suppress inflammatory cytokine release and improve cardiac outcomes in experimental sepsis models (19). However, our study extends existing literature by quantitatively demonstrating cytokine suppression across both Gram-positive and Gram-negative sepsis models, hence strengthening translational relevance.

Mitochondrial dysfunction has emerged as a critical driver of SIMI, linking inflammation to energy failure, oxidative stress, and cardiomyocyte death. Previous studies have shown that sepsis induces mitochondrial swelling, cristae disorganization, and impaired oxidative phosphorylation in cardiac tissue (20, 21). Preservation of mitochondrial integrity has therefore been proposed as a key therapeutic target in septic cardiomyopathy (22).

In agreement with earlier reports, we observed severe mitochondrial ultrastructural damage in septic myocardium, including cristae disruption and vacuolization. Notably, Xiangdan injection markedly preserved mitochondrial architecture, as evidenced by significantly reduced Flameng scores. This ultrastructural evidence provides direct morphological confirmation of mitochondrial protection, which has been inconsistently addressed in prior studies relying primarily on biochemical or functional assays (23). Our findings thus provide a mechanistic bridge between inflammation suppression and improved myocardial energetics in sepsis.

Although the present study did not directly investigate molecular signaling pathways, several mechanisms may explain the observed cardioprotective effects of Xiangdan injection. Previous studies have suggested that traditional Chinese medicine–derived formulations can modulate key inflammatory signaling pathways involved in sepsis, particularly the Toll-like receptor 4 (TLR4)/nuclear factor-κB (NF-κB) axis, which plays a central role in cytokine amplification and myocardial inflammation (12, 19). Suppression of this pathway may reduce the excessive production of pro-inflammatory mediators such as IL-6, TNF-α, and IL-1β (15–17). In addition, mitochondrial protection observed in this study may be related to improved mitochondrial homeostasis and reduced oxidative stress. Experimental evidence indicates that preservation of mitochondrial integrity during sepsis may involve regulation of mitochondrial quality-control pathways, including mitophagy-related mechanisms such as PINK1/Parkin signaling and attenuation of reactive oxygen species generation (20–23). These mechanisms may collectively contribute to improved mitochondrial function, reduced oxidative injury, and enhanced cardiomyocyte survival during septic stress.

Serum biomarkers such as BNP, CK-MB, and cTnI are widely used indicators of myocardial stress and injury in septic patients and have been independently associated with increased mortality (24, 25). In experimental models, elevated cardiac biomarkers correlate with histological myocardial damage and mitochondrial dysfunction (26).

In this study, Xiangdan injection significantly reduced all measured cardiac biomarkers in a dose-dependent manner. Importantly, these biochemical improvements were paralleled by reduced histopathological injury and preserved mitochondrial ultrastructure, indicating that biomarker normalization reflects genuine myocardial protection rather than transient hemodynamic effects. This integrative structural–biochemical correlation strengthens the validity of our cardioprotective conclusions.

A notable strength of this study is the use of both Gram-positive (*S. aureus) and Gram-negative* (*E. coli*) sepsis models. While most preclinical studies rely on a single sepsis model, clinical sepsis is etiologically heterogeneous. Prior reports have suggested that host inflammatory and myocardial responses may differ depending on pathogen type (27). Our data demonstrate that Xiangdan injection exerts comparable cardioprotective effects across both bacterial etiologies, suggesting that its therapeutic action targets host-response pathways rather than pathogen-specific mechanisms.

Xiangdan injection is derived from traditional Chinese medicinal herbs that have long been used for cardiovascular and circulatory disorders. Previous pharmacological studies have reported that its bioactive components exhibit anti-inflammatory, antioxidative, and microcirculatory regulatory properties, which are particularly relevant in the context of sepsis-induced organ injury. These compounds have been shown to modulate inflammatory signaling pathways, suppress excessive cytokine release, and improve endothelial and mitochondrial function in experimental models of cardiovascular injury. In addition, several studies suggest that traditional Chinese medicine formulations with similar compositions may regulate oxidative stress responses and mitochondrial homeostasis, thereby protecting cardiomyocytes from inflammatory and oxidative damage. These pharmacological characteristics provide a plausible explanation for the observed reductions in inflammatory cytokines, cardiac injury biomarkers, and mitochondrial ultrastructural damage in the present study, supporting the potential therapeutic value of Xiangdan injection in sepsis-induced myocardial injury (12–14).

An important contribution of the present study lies in the systematic validation of Xiangdan-mediated myocardial protection across both Gram-positive and Gram-negative models of experimental sepsis. While previous studies have reported anti-inflammatory and cardioprotective effects of traditional Chinese medicine formulations, most investigations have relied on a single sepsis model. By employing both Staphylococcus aureus and Escherichia coli models, the present study demonstrates that the protective effects of Xiangdan injection are largely independent of bacterial pathogen type. In addition, the use of transmission electron microscopy to directly visualize mitochondrial ultrastructure provides morphological evidence of mitochondrial preservation during septic injury. These findings strengthen the mechanistic link between inflammatory suppression and mitochondrial protection in sepsis-induced myocardial injury.

### Limitations and future directions

Several limitations of the present study should be acknowledged. First, direct assessment of cardiac function was not performed. Although histopathological injury scores, cardiac injury biomarkers, inflammatory cytokines, and mitochondrial ultrastructural analysis provide important indicators of myocardial injury, these parameters cannot fully substitute for functional cardiac measurements such as echocardiography or hemodynamic analysis. Future studies incorporating *in vivo* cardiac functional assessment will therefore be necessary to more comprehensively evaluate the cardioprotective effects of Xiangdan injection in sepsis.

Second, Xiangdan injection was administered prior to and immediately after sepsis induction, representing a preventive experimental design intended to evaluate the ability of the compound to modulate early inflammatory and mitochondrial injury processes during the acute phase of sepsis. However, this regimen does not fully reflect clinical treatment conditions, where therapeutic interventions are typically initiated after sepsis diagnosis. Future studies should therefore investigate the therapeutic efficacy of Xiangdan injection when administered after sepsis onset in order to better assess its clinical translational potential.

Third, the present study focused on the acute phase of sepsis, with experimental endpoints evaluated at 12 h after bacterial challenge. Although this time point effectively captures early inflammatory responses and mitochondrial injury, the progression of sepsis-induced myocardial injury and its functional consequences may continue to evolve over longer periods. Future studies incorporating extended observation periods and survival analysis beyond 24–72 h will be necessary to evaluate longer-term cardioprotective effects.

Finally, although the present study demonstrated significant suppression of inflammatory cytokines and preservation of mitochondrial ultrastructure, the molecular mechanisms underlying these protective effects were not directly investigated. Potential pathways may involve modulation of inflammatory signaling cascades such as the TLR4/NF-κB pathway, regulation of oxidative stress responses, or mitochondrial quality-control mechanisms including mitophagy-related pathways such as PINK1/Parkin signaling. These mechanisms remain speculative and require further investigation using molecular approaches including Western blotting, gene expression analysis, and pathway-specific inhibition studies.

### Clinical implications

Despite these limitations, the present study provides compelling experimental evidence that Xiangdan injection offers multi-level myocardial protection during sepsis. By simultaneously suppressing excessive inflammatory responses and preserving mitochondrial ultrastructure, Xiangdan injection targets two central and interrelated mechanisms of SIMI. These findings not only extend current understanding of traditional Chinese medicine–based interventions in sepsis but also support further translational and clinical investigations of Xiangdan injection as an adjunctive therapy for sepsis-related cardiac dysfunction.

## Conclusion

The present study demonstrates that Xiangdan injection exerts early cardioprotective effects in murine models of experimental sepsis induced by both Gram-positive and Gram-negative pathogens. Xiangdan administration improved survival at the 12-hour acute phase, attenuated myocardial structural injury, suppressed systemic inflammatory responses, reduced circulating cardiac injury biomarkers, and preserved myocardial mitochondrial ultrastructure in a dose-dependent manner. The coordinated improvement in inflammatory, biochemical, histopathological, and ultrastructural parameters suggests that Xiangdan injection mitigates early sepsis-induced myocardial injury through combined modulation of inflammatory responses and preservation of mitochondrial integrity. Although the present findings primarily reflect the acute phase of septic injury, they provide experimental evidence supporting the potential cardioprotective role of Xiangdan injection in sepsis. Further studies incorporating extended observation periods, functional cardiac assessments, and detailed molecular investigations are warranted to elucidate the underlying mechanisms and evaluate the translational potential of this therapeutic strategy.

## Data Availability

The original contributions presented in the study are included in the article/supplementary material, further inquiries can be directed to the corresponding author.
